# Miniature brain encodes optomotor response behavior in tiny thrips

**DOI:** 10.1016/j.isci.2026.114966

**Published:** 2026-02-10

**Authors:** Tomer Urca, Fritz-Olaf Lehmann

**Affiliations:** 1 Department of Animal Physiology, Institute of biological Sciences University of Rostock, Albert-Einstein-Str. 3, 18059 Rostock, Germany

**Keywords:** Molecular neuroscience, Developmental neuroscience, Systems neuroscience

## Abstract

Miniaturization of insects requires a reduction and simplification of morphological and physiological systems. An extreme example is the visual system of tiny thrips that has only 120 ommatidia and a tiny brain for image processing. To investigate the significance of this reduction, our study combines measurements of optical properties of ommatidia using micro-tomography with a behavioral assay on optomotor response and numerical analyses on the movement detector. We found large interommatidial and photoreceptor acceptance angles of ∼18°. During stimulation by a rotating stripe pattern, thrips walk on circular paths to compensate for retinal slip. We estimated ∼3.5 Hz stimulus temporal frequency for maximum optomotor response and ∼41 ms time constant for the low-pass filter of the movement detector. Our study suggests that even animals with an extreme miniaturization of their sensory systems maintain optomotor behaviors and possibly neural coding strategies of visual information that are typical for larger insects.

## Highlights

• Tiny brain and small number of ommatidia limit vision in miniature thrips
• Object recognition is hampered by large interommatidial and acceptance angles of 18°
• Thrips show vision-mediated optomotor behavior even at reduced visual capacity
• The behavior results from elementary motion detection with a 41 ms temporal delay

## Graphical abstract

**Figure:**
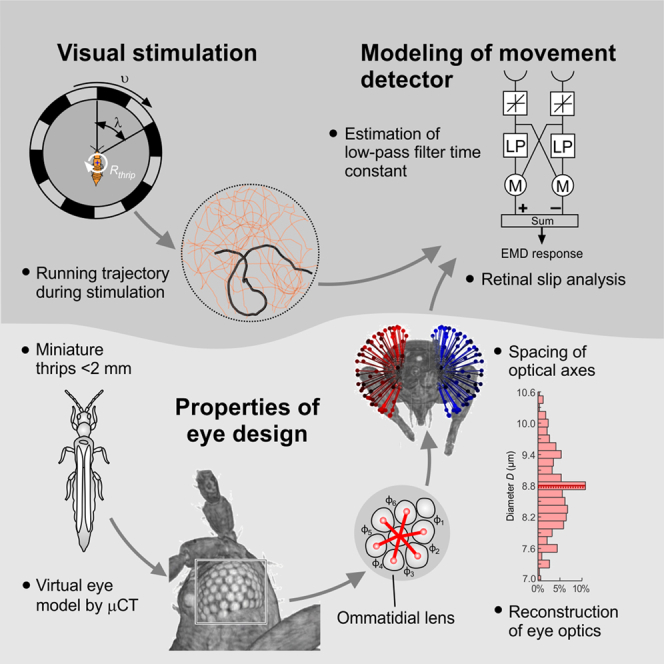

## Introduction

Optomotor response is a visually mediated behavior that enables insects to maintain body posture stability and course control during locomotion.^1^ The reflexive optomotor action is triggered by the surrounding visual perception of motion and prompts compensatory head and body movements in order to stabilize retinal optic flow.^2^^,^^3^^,^^4^ Thus, when an insect detects movements of its surrounding, it instinctively adjusts its body position by turning in the same direction as the perceived motion.^5^ This behavior thus helps to counteract unintended body drifts and to avoid collisions with obstacles.^6^^,^^7^^,^^8^ The response is initiated during locomotion, such as flight or walking, and its strength depends on the properties of the perceived optic flow. Main properties are, for example, spatial wavelength and temporal frequency of the moving visual pattern. It is, however, the particular optics and visual processing power of the insect’s visual system that defines how these parameters are perceived and translated into a behavioral response.

Numerous, previously published studies suggest that the optomotor response can be predicted by the numerical elementary motion detector (EMD) model, which integrates several properties of the underlying neural visual ganglia.^9^ The correlation-based EMD model involves two spatially separated photoreceptors or ommatidia and their average divergence (interommadial angle) and acceptance angles. The visual signal collected by each ommatidium is subject to temporal filtering (temporal delay), creating a delayed version of the signal, which is then multiplied with the instantaneous signal from a neighboring ommatidium. This signal is subtracted from the product of other pairs of signals with an opposite temporal delay, resulting in a directional motion signal.^10^ Motion direction and speed are computed by a neural network integrating local motion signals across the visual field. Small interommatidial angles and acceptance angles produce little image overlap between photoreceptors, leading to higher spatial resolution and wavelength sensitivity. Shorter temporal delays yield higher sensitivity to changes in temporal frequency.^6^^,^^11^

The sensitivity of the visual system decreases with decreasing body size as smaller eyes support fewer ommatidia. Small corneal lenses and apertures cause elevated light diffraction and limit the number of photons available to the rhabdomers.^12^^,^^13^ As a consequence, small eyes have lower signal-to-noise ratios at the level of the photoreceptors, including their downstream neurons.^14^ Both factors, light diffraction and low signal-to-noise ratio, attenuate the eye’s resolving power and thus the detectability of small visual objects at low light conditions. In addition, small brains limit the computational power available for visual processing. This is taken to the extreme in miniature insects such as the 1.0 mm (∼52.9 μg body mass^15^) oriental flower thrips *Frankliniella occidentalis*, whose tiny, rounded eye contains only 60 ommatidia.^16^ Similar values are reported for the smallest known insect species, *Megaphragma mymaripenne* (Hymenoptera), with only 30 ommatidia on each body side and for Strepsiptera with typical 20–50 ommatidia in each eye.^17^^,^^18^ In the latter group, *Tridactylophyagus similis* has as few as 10 ommatidia, the round eye of *Xenos verparum* 10–15 and *Stylops muelleri* as many as 150.^19^^,^^20^ A recent study on the tiny whitefly *Bermesia tabaci* reported 73 ommatidia on each body side.^21^ These are a miniscule numbers compared, for example, to the ∼303 ommatidia in ∼1.5 mm fungus gnat *Neoplatyura modesta*,^22^ ∼782 ommatidia in each eye of the 3.0 mm fruit fly,^23^ ∼5000 ommatidia in the ∼20 mm worker honeybee^24^ and over 30,000 in large dragonflies.^25^ To allow a reasonable panorama view, round eyes of miniature Hymenopterans and Coleopterans typically have large divergence angles of more than ∼15° - a value several-fold higher than in most larger insects.^26^^,^^27^

Miniaturization also limits the number of brain cells for sensory processing and motor control.^18^^,^^28^^,^^29^^,^^30^ Smallest insects of the groups of Mymaridae and Trichogrammatidae even lost a large number of cell bodies of their neurons, and up to ∼97% volume of their brain is neuropil.^31^ It is also shown that tiny Strepsiptera have cell bodies at the lower size limit of ∼2 μm diameter.^28^ Estimations based on histology suggest that the brain of thrips comprises only ∼10,000 brain cells^32^ that is ∼20-fold less than the ∼200,000 neurons^33^ in *Drosophila*. Assuming 21 neurons for each ommatidium for visual motion processing (*Drosophila*), ∼25% of all neurons in the thrips would be needed for motion analysis compared to ∼17% in the fruit fly.^34^ Moreover, little is known about the behavioral capacity of small brains. For example, previously published studies on eye sight in the *thrips tabaci* cryptic species, including *Frankliniella occidentalis,* focused on contrast intensity and spectral sensitivity in experiments that attract animals to stationary visual targets.^35^^,^^36^^,^^37^^,^^38^ Although a detailed study on tethered parasitic Strepsiptera *Xenos verparum* showed head and abdomen deflection when the animal was passively rotated inside a visual panorama, data on optomotor response behavior during locomotion in miniature insects are rare.^17^

To estimate the visual capacity and the existence of optomotor behaviors in a miniature insect, including vital values for the elementary motion detector (EMD) model, we here combined measurements of optical properties in *F. occidentalis* based on micro-tomography (μCT) images with a behavioral assay on vision-controlled walking. The walking paths are scored at multiple velocities and object sizes of the visual stimulus. From walking path data and lens optics, we eventually approximated the time delay of the numerical motion detector model, estimated the stimulus temporal frequency for maximum optomotor response, and compared the EMD output with the measured optomotor responses.

## Results and discussion

### Optical properties of ommatidia

**Figure 1:**
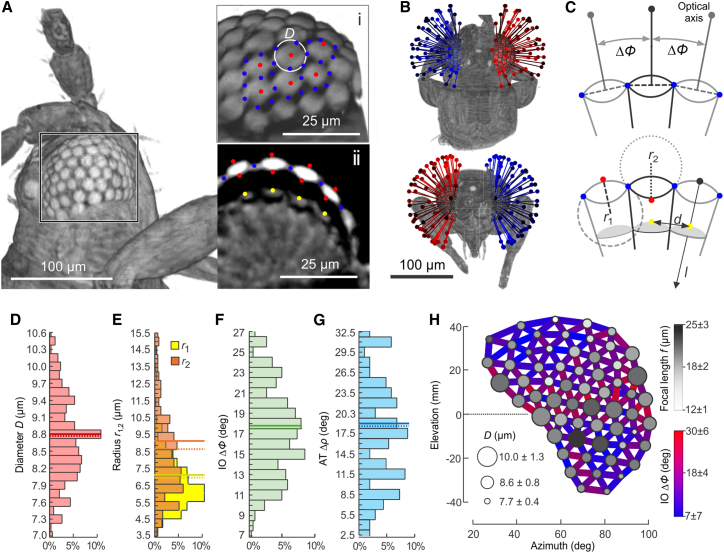
Optical apparatus of the thrips *Frankliniella occidentalis* (A) Micro-tomographical reconstruction of prothorax and head (side view). Ommatidia in the region of interest (box) is shown enlarged in *i* and a cross section of the eye in *ii*. Red dots mark the outer and inner lens tips (red), lens perimeter (blue) and photoreceptors (yellow). (B) Mean gaze direction (optical axes) of all ommatidia in the left (blue) and right (red) eye overlaid on μCT-model of the eye. Each vector is orientated normally to the lens base. View is from dorsal (top image) and frontal (bottom image). (C) Optical lens parameters. Δ*ϕ*, interommatidial angle (IO); *d*, photoreceptor distance between neighboring ommatidia; *D*, mean lens diameter; *l*, focal length of an ommatidium; *r*_1_, inner lens radius; *r*_2_, outer lens radius. (D–G) Histograms of optical parameters showing lens diameter derived from the blue markers shown in A image ii in D, mean lens radius in E, interommatidial angle (IO) in F and lens acceptance angle (AT) in G. Means, solid line; medians, dotted line; *n* = 299 ommatidia in D–F and *n* = 185 in G, *N* = 5 heads. (H) Two-dimensional projection map of the right eye (means, *N* = 5 animal heads) in spherical elevation and azimuth coordinates. Each circle represents a single ommatidium, circle size is lens diameter and circle gray value focal length. Interommatidial angles are encoded in pseudo-color of lines connecting two circles each.

We scored optical properties of 299 ommatidia from five thrips heads that were 3-dimensionally reconstructed by micro-tomography (Figure 1). Mean lens diameter is comparably large and amounts to ∼8.76 ± 0.76 μm (means ± standard deviation, *N* = 299, Figure 1). This value compares to lens diameters of ∼17.4 μm in the fruit fly *Drosophila mauritiana*, ∼20.7 μm in the honeybee *Apis mellifera*, ∼28.2 μm in the moth *Deilephila elpenor,* and ∼32.0 μm in the ∼1.5 g moth *Manduca sexta*.^39^^,^^40^ Outer and inner lens height as measured from the lens principal plane is ∼1.61 ± 0.37 μm and ∼1.28 ± 0.48 μm, respectively (Figure 1C), and thus outer and inner radii of curvature ∼7.10 ± 1.52 μm and ∼9.48 ± 4.38 μm, respectively (Figure 1E). Focal length of the lens depends on the radii-normalized ratio of refraction between the transition from air to lens cornea and from lens proteins to crystalline cone proteins (cf. method details section). The above radii translate to a mean focal length of ∼20.2 ± 6.78 μm or 2.3-fold the lens diameter (Figure 1H). The ommatidia’s field of view determines optical resolution and spacing of the optical axes; the eye’s is thus central for panorama view.^21^ We found that mean angular spacing (interommatidial angle, IO, Δ*ϕ*) is ∼17.8° ± 4.43° (Figure 1F), covering large parts of the environment but hampering visual resolution and thus likely visual motion detection. For comparison, the dorsal and ventral eye sections of the similar-sized whitefly *Bermesia tabaci* have slightly smaller interommatidial angles of ∼12.5 and ∼16.2, respectively.^21^ A slightly higher value of 19.2° was found for the eye of the miniature strepsipteran species *Xenos vesparum*.^17^ Lens diameter, focal length, and interommatidial angle are not homogenously distributed in the thrips eye. The smallest values we found for the flattened dorsal and ventral eye sections, and the largest about the mid horizontal of the eye (Figure 1H). The changes are approximately ∼1.4–∼4.3-fold and range from ∼7.7 ± 0.4 μm to ∼10.9 ± 1.3 μm, ∼12.0 ± 1.0 μm to ∼25.0 ± 3.0 μm and ∼7.0° ± 7.0°–∼30.0° ± 6.0°, respectively (Figure 1H). The significance of the latter variances is unknown.

The small animal size and limited resolution of microCT compared to SEM make it difficult to precisely estimate the spacing between the rhabdomen of the photoreceptor cells and thus to calculate the acceptance angle (AT, Δ*ρ*) of an ommatidium. The acceptance angle determines the spatial frequency that is transmitted to the optical system and the ratio between the photoreceptor distance and the focal length of the cornea. The angle is a critical value and typically varies between ∼0.5 and ∼2 times the ommatidial angle in insects.^13^^,^^41^^,^^42^ The latter value is also predicted for optimal sampling.^13^ In *X. vesparum* te acceptance angle was approximated as ∼53.9°, which is ∼2.8-fold the interommatidial angle.^17^ In *F. occidentalis,* we estimated bona fide ∼5.79 ± 0.93 μm mean distance between neighboring photoreceptors (*N* = 120 ommatidia from 2 animals, Figure 1A ii). This measure converts into a visual acceptance angle of ∼17.7° ± 1.14° (*N* = 185 ommatidia, Figure 1G). The acceptance angle in thrips is thus similar to the interommatidial angle, which is typical for diurnal insects. Comparable ratios (Δ*ρ*/Δ*ϕ*) are reported for other insects, such as the praying mantis *Tenodora australasiae* (∼1.14),^43^ the robberfly *Holcocephala fusca* (∼1.04),^44^ the European wasps *Vespula germanica* and *V. vulgaris* (∼1.04),^45^ honeybee drones *Apis mellifera* (∼0.85),^46^ bumblebee *Bombus* sp. (∼0.71),^47^^,^^48^ butterflies spp. (∼1.0),^49^^,^^50^ the killerfly *Coenosia attenuata* (∼1.31),^23^ and the fruitfly *Drosophila melanogaster* (∼1.78),^23^ despite the ∼2.2-times (fruitfly) to ∼26-times (mantis) larger acceptance and interommatidial angles in the thrips.

### Behavioral response to visual stimulation

**Figure 2:**
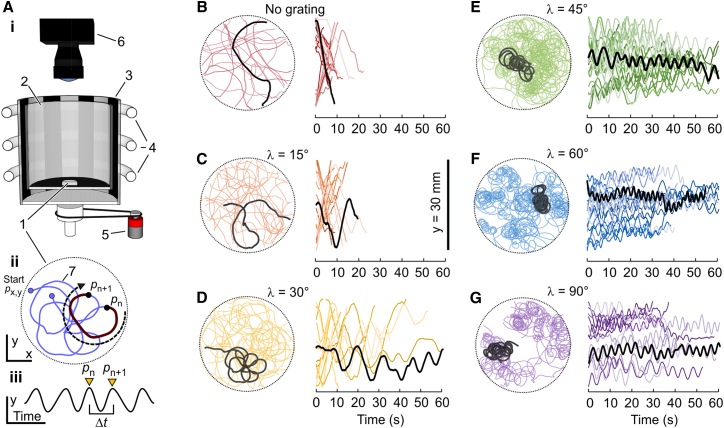
Optomotor response in walking thrips (A) Experimental setup. Thrips are walked in an arena (3 cm diameter) surrounded by a rotating visual pattern in *i*. Example of a walking path that highlights one full body rotation (black) between positions *p*_n_ and *p*_n+1_ in *ii*. The y-position of the animal in *ii* plotted against running time in *iii*. Inflection points *p*_n_ and *p*_n+1_ of the time trace determine the period Δt for 360° body rotation. 1, walking platform; 2, inner stationary translucent plexiglas cylinder; 3, rotating pattern cylinder with frosted surface and printed stripe pattern; 4, circular fluorescent light tubes for backlight illumination; 5, electrical gear motor for pattern rotation; 6, high speed video camera. Drawing is not to scale. (B–G) Walking path trajectories and time traces of y-position of all tested animals (color-coded). Temporal frequency of pattern rotation is 3.5 Hz. For each wavelength, running trace of a single animal is emphasized in black. Number of running traces are *N* = 24, 23, 18, 26, 21, 15 in B–G, respectively.

**Figure 3:**
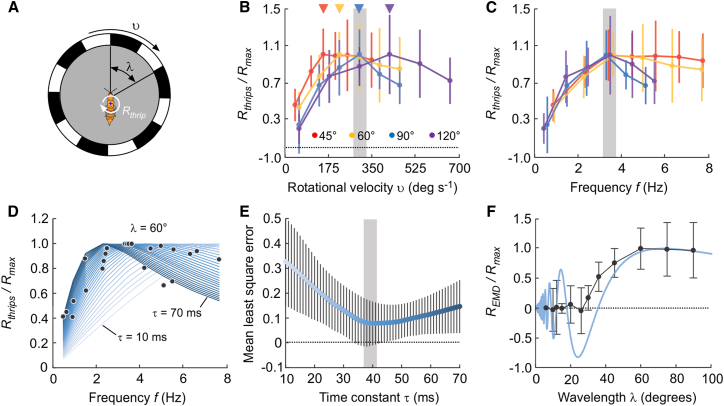
Behavioral response and numerical simulation depending on stimulus velocity and wavelength (A) Visual parameters and behavioral response. (B) Normalized optomotor response (*R*_thrips_/*R*_max_) of the tested animals during stimulation at *λ =* 45°, 60°, 90° and 120° pattern wavelength and plotted against rotational velocity *υ* of the visual pattern. Arrows indicate measured peak responses. Means ± standard deviation; *N* = 21, *N* = 11, *N* = 15 and *N* = 8 animals, respectively. (C) Same data as shown in B but plotted against temporal frequency *f* of the visual stimulus. Gray bar indicates best (maximum) response. Means ± standard deviation. (D) Normalized EMD-response curves dependent on different delays of the EMD temporal filter (*τ* = 10 ms [pale] – 70 [dark] ms, 2.4 ms step size) plotted against temporal frequency of the visual stimulus with *λ* = 60°. Superimposed black dots are the measured mean responses as shown in B (*N* = 24 running traces). (E) Best fit analysis of time constant while varying stimulus frequency. Least square error between each EMD response curve shown in D and behavioral response measured in *F. occidentalis* shown in C. Color encodes time delay of the EMD model (*τ* = 10 ms [pale] – 70 [dark] ms, 1.2 ms step size; means ± standard deviation, *N* = 50 time constants). (F) Wavelength sensitivity analysis of *F. occidentalis’s* visual system measured as mean normalized response for wavelengths ranging from 6° to 90° rotating at 3.5 Hz optimal frequency (*f*_opt_). Thrips optomotor response is shown in black (means ± standard deviation) and simulated EMD response in blue. *N* = 24 (6°), *N* = 25 (10°), *N* = 21 (12°), *N* = 23 (15°), *N* = 13 (20°), *N* = 15 (26°), *N* = 18 (30°), *N* = 8 (36°), *N* = 21 (45°), *N* = 11 (60°), *N* = 21 (75°) and *N* = 15 running traces (90°).

To test thrips on their ability to elicit optomotor response behaviors, the animal freely walked in an arena surrounded by a rotating grating consisting of black and white stripes (Figures 2A and 3A). To determine the animal’s sensitivity to the motion stimulus, we varied the angular velocity and spatial wavelength of the grating and recorded the locomotor behavior for 68 s after placing the thrips inside the experimental setup. Arena rotation velocity was verified beforehand (Figure S1). Figure 2B shows that in a seemingly stationary environment (“rotation with no grating”), the animals walk mostly straight or at comparatively large path curvatures for up to ∼20 s before they rest. We did not find a bias toward a specific direction inside the arena. Approximately 1.76 ± 1.0 s (*N* = 26 runs) after the onset of drum rotation, the animals change their behavior by walking in circular paths, as is typical for optomotor response. The latter value is similar to the ∼1.5 s delay of head movements during passive body rotation of *X. vesparum* until the response reaches a plateau during passive rotation.^17^ Figures 2C–2G shows that with increasing spatial wavelength of the visual pattern from 15° to 90° at a constant temporal frequency of ∼3.5Hz, the radius of the circular paths typically decreases. We did not attempt to quantify the diameter of the path circles in greater detail, but derived the strength of optomotor response (*R*_thrips_) from the turning velocity of the animal. By decomposing the animal’s x- and y-body position inside the arena, turning velocity is the time between two positive peaks of the animal’s y-position time trace divided by 360° to complete a full walking circle (cf. method details section, Figures 2A and 3). Deviations in stimulus wavelength due to the ±1.5 cm walking range inside the arena were negligible and not further considered in the analyses (Figure S2).

Assuming that the optomotor behavior of the tested thrips relies on a Reichardt elementary motion detector,^11^ the thrips’ optomotor response should depend on the temporal frequency of the visual pattern and thus on the ratio between angular velocity and spatial wavelength. We thus systematically stimulated the animals with various combinations of 6–7 angular velocities ranging from ∼30 to ∼700 deg s^−1^ and 4 spatial wavelengths (45°, 60°, 90°, and 120°, Figure 3B). Figure 3A shows that changes in angular velocity produce tuning curves for normalized optomotor responses (*R*_thrips_/*R*_max_) with a maximum at ∼150°s^−1^ (45°), ∼215°s^−1^ (60°), ∼296°s^−1^ (90°), and ∼419°s^−1^ (120° spatial wavelength). Non-normalized turning velocity of the animals is shown in supplemental data Figure S3. Maximum response peaks converge at ∼3.5 Hz temporal frequency (*f*_opt_) for all tested spatial wavelength that likely supports the assumption of motion processing by a conventional elementary motion detector network (Figure 3C). A slightly smaller *f*_opt_ at ∼2.2 Hz stimulus spatial wavelength was reported for the head response in immobilized *X. vesparum*.^17^

### Modeling properties of optomotor responses and wavelength sensitivity function

The output of Reichardt’s correlation-based elementary motion detector (EMD) for visual motion detection in insects depends on several parameters, including spatial wavelength *λ* and angular velocity υ of the visual stimulus, Δ*ϕ* interommatidial and Δ*ρ* acceptance angles of the optical apparatus, and the temporal low-pass filter time constant *τ* of the neural network for motion detection.^51^^,^^52^ The results in Figures 1, 2, and 3 provide these data except for the time constant. However, the EMD time constant can be derived by fitting the output of the EMD model (*R*_*EMD*_) to our experimental data (*R*_*thrips*_). A simplified version of a numerical EMD model can be written as:

Equation 1 $$R_{EMD}={\left(1+{\left[2\pi \tau \upsilon /\lambda \right]}^{2}\right)}^{-0.5}\times \sin\left(\arctan\left[2\pi \tau \upsilon /\lambda \right]\right)\times \sin\left(2\pi \Delta \phi /\lambda \right)\times {\left(1+{\left[\Delta \rho /\lambda \right]}^{2}\right)}^{-0.5}$$

where the first term is the first-order low-pass filter of the EMD, the second the temporal frequency term, the third term the so-called interference term, the fourth the optics term, and υ/*λ* is the temporal frequency *f* of the stimulus.^17^ We approximated the missing EMD time constant of the thrips by comparing the EMD output with the maximum optomotor response from our experiments (*f*_opt_ = ∼3.3–∼3.7 Hz, Figure 3B) using a least square error (LSE) fit approach. This was achieved by the insertion of the morphological parameters into Equation 1 and testing various time constants from 10 ms to 70 ms (1.2 ms step size) at 60° spatial wavelength depending on the temporal frequency of the stimulus. The procedure produces a swarm of optomotor response curves that peak depending on the selected parameters (Figure 3D). Figure 3E shows that at 60° pattern wavelength, LSE is minimum (∼0.08) at *τ* = ∼41 ms but varies only a little (±0.008 change in LSE) between ∼36 ms and ∼50 ms. A slightly higher time constant of ∼70 ms has been reported for the visual system of tiny *X. vesparum*.^17^ For a first-order low-pass filter, the theoretical time constant for EMD is 1/(*2πf*_opt_), which amounts to ∼45 ms for *f*_opt_ = ∼3.5Hz.^53^ The small difference between measured (∼41 ms) and predicted (∼45 ms) values might be due to motion adaptation of motion integrating neurons. For example, the time constant of H1 neuron activity in the blowfly *Calliphora vicina* is ∼300 ms in the unadapted state and less than ∼30 ms after adaptation to a visual motion stimulus.^54^ This suggests a 10-fold dependency on the internal state of the EMD neural network. Recordings in HS-cells of the dronefly *Eristalis tenax* suggest that motion stimulation of 4 s is sufficient for the adaptation process.^55^ For comparison, Figure 2 shows that we scored an up to ∼60 s behavioral response time in the tested thrips. Nevertheless, alternative explanations assign the above finding to the difference in image step and continuous stimulation responses.^55^ Notably, the optimal temporal frequency in thrips for optomotor response is in the range of the values reported for walking *Drosophila melanogaster*. This is surprising because the much larger fruit fly has more than ten times the cerebral neurons and ommatidia of a miniature insect and a small 4° interommatidial angle.

Eventually, we calculated the thrips’ wavelength sensitivity function using the EMD model and compared its outcome to the experimental data. This comparison is shown in Figure 3F using a temporal frequency of 3.5 Hz for numerical modeling (see Figure S3B for non-normalized data). At low wavelength, the model output shows strong fluctuation from spatial aliasing or geometric interference between the period pattern and the EMD input array, which is typical for the model.^17^ Our data show that the optomotor response of behaving thrips is minimum at wavelengths between ∼6° and ∼26°, increases with increasing wavelength until ∼50°, and approximately saturates at wavelengths above ∼60° (Figure 3F). Notably, the maximum theoretical output of correlation-based EMD is predicted at *λ*_*max*_ *=* 4Δ*ϕ*. In the thrips, mean Δ*ϕ* is ∼17.8° ± 4.43° (*N* = 299 ommatidia) that converts into ∼*λ*_*max*_ = 72 ± 16°, a value that is close to the saturation point of our measured wavelength sensitivity curve (Figure 3F). A similar large *λ*_*max*_ value had been predicted for deflection angles of head and abdomen ranging from ∼60° to ∼70° in tethered *X. vesparum* during stimulation with a stripe grating.^17^ For comparison, larger insects such as *Drosophila melanogaster* have more than ten times the brain neurons and ommatidia of miniature insects and an interommatidial angle of ∼4° across the entire eye. Nonetheless, the thrips' maximal response is elicited in the range of optimal temporal frequencies reported for walking *Drosophila*.^56^^,^^57^^,^^58^

In conclusion, miniaturization of an animal leads to considerable challenges, hampering almost all organs and structures. In insects, miniaturization leads to simplifications of digestive and excretory systems, dropping crops and gizzards, circulatory systems by losing vessels and pulsatile organs, and a reduction in tracheal branching.^18^ Interestingly, the muscular system is highly conserved in miniature insects compared to larger relatives, while sensory systems are often dramatically reduced in the number of receptors, such as sensilla on the antennae or ommatidia of the eye. The central nervous system for sensorimotor control is concentrated but still differentiated, similar to larger species, and its asymmetry is a peculiar feature in miniature insects.^18^ The goal of this study was to highlight how miniature insects maintain vision-guided locomotor behaviors with such a reduced set of morphological structures. Our data suggest that optomotor responses by movement detection remain a necessary part of the behavioral repertoire even in a species that is plant-sucking and a weak flyer dispersed passively by wind.^59^^,^^60^^,^^61^ Our data also provide evidence that the computational rules for motion processing in tiny species might be similar to those in larger insects. Altogether, the above findings raise the question of why miniature insects such as the thrips kept optomotor behaviors during evolution. The factors that limit the minimum size of a complex animal are still under debate, but often focused in the light of its morphological structures and little on their physiological function, which needs further investigations. Besides their morphological and physiological adaptations, miniature insects are, moreover, model systems for locomotion covering wing aerodynamics and flight muscle energetics and thus represent a group of animals suitable for integrative studies at the lower limit of body sizes.^62^

### Limitations of the study

The 3-dimensional virtual models of the insect’s visual apparatus are reconstructed using micro-computer tomography. Compared to high-resolution scanning electron microscopy, micro-computer tomography has limited optical resolution, which lowers the accuracy of the reported optical parameters. As thrips are not the smallest insect species, our findings should be confirmed on a broader level in other miniature insects.

## Resource availability

### Lead contact

Further information and requests for resources and methods should be directed to the lead contact, Fritz-Olaf Lehmann (fritz.lehmann@uni-rostock.de).

### Materials availability

Animals are available from the lead contact upon request.

### Data and code availability

The authors declare that the data supporting the findings of this study are available within the article.

• Data: All data reported in this article will be shared by the lead contact upon request.
• Code: This article uses self-developed software (Calculation.m) written in MATLAB. The download link for the software is provided in the key resources table.
• Other items: Any additional information required to understand the results is available from the lead contact upon request.

## Acknowledgments

We thank Christian Wirkner and Stephan Scholz (Institute of Life Sciences, Department of General and Special Zoology, 10.13039/501100012688University of Rostock, Germany) for their help on micro-tomography imaging. This work was supported by the 10.13039/501100001659Deutsche Forschungsgemeinschaft (10.13039/501100001659DFG) by grant LE 905/18-1 to F.O.L.

## Author contributions

Conceptualization: T.U.; methodology: T.U. and F.O.L.; formal analysis: T.U.; investigation: T.U.; resources: F.O.L.; data curation: F.O.L.; writing - original draft: T.U.; writing - review and editing: F.O.L.; visualization: T.U.; project administration: F.O.L. and T.U.; funding acquisition: F.O.L.

## Declaration of interests

The authors declare no competing interests. Current affiliation of Tomer Urca is Experimental Zoology Group, University of Wageningen, 6708WD Wageningen, The Netherlands.

## STAR★Methods

### Key resources table

**Table:** 

REAGENT or RESOURCE	SOURCE	IDENTIFIER
**Chemicals**		

Duboscq-Brasil fixative	This study	–

**Experimental models**		

*Frankliniella occidentalis* (wildtype)	This study	–

**Software and algorithms**		

Calculation.m (Matlab)	This study	https://doi.org/10.5281/zenodo.15352423
ImageJ	ImageJ	https://imagej.net/ij/
Matlab® 2018b	The Mathworks	https://mathworks.com
Powerpoint 2016	Microsoft	https://www.microsoft.com
CoreDRAW X6	Corel Corporation	https://www.coreldraw.com
DLTdv7	Tyson Hedrick	https://biomech.web.unc.edu/dltdv/
Vaa3D	Hanchuan Peng	https://github.com/vaa3d

**Other**		

Xradia 410 Versa μCT	Zeiss	https://www.zeiss.com/microscopy
EM CPD300 Point dryer	Leica	https://www.leica-microsystems.com

### Experimental model and study participant details

The western flower thrips *Frankliniella occidentalis* (Pergande) used in this study are listed in the key resources table and include both male and female organisms at unknown age. Both sexes are similar regarding eye structure and thus there is no influence of sex on the presented results. The wildtype animals stem from our laboratory stock and were initially provided by Gal Ribak, Tel Aviv University, Israel.

### Method details

#### Animal rearing

The animals were reared within a 70 cm × 70 cm × 100 cm tent at the Department of Animal Physiology, University of Rostock. They were raised on sweet potato, eggplant and pumpkin plants and kept under constant environmental conditions at 27±3°C, 50% – 70% relative humidity and a 13h/11h day/night cycle. For testing, we collected thrips with an aspirator, anesthetized them on a 1°C Peltier stage and kept them in small containers for micro-tomographic preparation or behavioral testing.

#### Micro-tomography

As this study required 3-dimensional models of the thrips head, we used micro-tomography instead of high-resolution 2-dimensional scanning electron microscopy. For x-raying, we removed head and thorax from the abdomen and transferred the torsi to glass vials containing Duboscq-Brasil fixative. After tissue fixation for 24 hours at room temperature, we dehydrated the torsi for 10 minutes each in rising concentrations of ethanol of 70%, 80%, 90%, 96% and 98.8 % (twice in each solution) and subsequently in a critical point dryer (Leica EM CPD300). The bodies were mounted inside a microCT (Zeiss Xradia 410 Versa X-Ray) at 6 mm distance from the X-ray source and 1 mm away from a 40x magnifying lens. We performed the scans at 50 kV and 8W, and 10 s exposure time per image without pixel binning. Image resolution is 0.29 μm x 0.29 μm per video pixel and images were exported as lossless TIF-stacks. During post-processing, we enhanced image contrast using ImageJ (Wayne Rasband, NIH).

#### Reconstruction of eye properties

We created 3-dimensional head models from 5 animals by uploading the microCT tiff stack files into Vaa3D software.^63^ Morphological markers for each ommatidium were manually digitized from the topography of the eye: one marker was on the highest and lowest points of the outer and inner rim of the lens, respectively, six markers indicated the lens base, and one marker was at the transition between crystal cone and rhabdomer (Figure 1A). The procedure followed a previously published approach on the visual system of miniature insects.^26^^,^^27^ Three additional markers on the tip of each ocellus defined a triangular plane for normalizing head orientation. We exported marker coordinates as an Excel file (Microsoft) and analyzed the data using a self-written code in MATLAB. The software included the following steps: (1) angular orientation of the head was normalized to zero angles, (2) markers of each lens were rotated so that the area of the lens base was maximum, (3) lens diameter *D* was estimated from the area of a circular function fitted to the lens base markers (blue, Figure 1A), and (4) orientation of the visual axis of each ommatidium was derived from the projection line through the digitized point on the lens top and the midpoint of the fitted circle (Figure 1B).

#### Optical properties of an ommatidium

The interommatidial angle (IO) is the angle between two neighboring visual axes (Figure 1C) and was estimated in a 2-dimensional plane between neighboring ommatidia (Figure 1H). For a thin lens with diameter *D*, the inner *r*_1_ and outer *r*_2_ radii of curvature of each corneal lens (spherical dome) can be written as:

Equation 2 $$r_{1,2}=\left(\frac{D^{2}}{4}+h^{2}\right)/2h$$

with *h* the half thickness of the lens, i.e. the distance of the lens tip from the lens principal plane.^64^^,^^65^ We estimated the focal length of each lens *l* from lens radii and refractive indices using the lens maker formula for thin lenses,^64^ written as:

Equation 3 $$\frac{1}{l}=\frac{n_{L}-n_{A}}{r_{1}}-\frac{n_{L}-n_{C}}{r_{2}}$$

with *n*_L_ the refractive index of the lens at the transition from air to the lens (*n*_L_=1.45),^27^ *n*_A_ the index of air (*n*_A_ = 1) and *n*_C_ the index from the lens proteins to the crystalline cone (*n*_C_=1.35).^66^ Indices *n*_L_ and *n*_C_ were adopted from the compound eye of the honeybee *Apis mellifera*.^66^ Acceptance angle of an ommatidium equals:

Equation 4 $$\Delta \rho =\frac{d}{f}$$

with *d* the mean distance between two neighboring rhabdomers (Figure 1C).

#### Behavioral assay

The animals were visually stimulated in an arena composed of two concentric 200 mm tall acrylic cylinders (Figure 2A). The inner cylinder had 140 mm diameter, was translucent and stationary, and surrounded by a 190 mm diameter rotating drum displaying the black stripe grating. The grating was printed on transparent plastic foil. The drum was illuminated from the back by three surrounding circular fluorescent light tubes behind a frosted surface to provide uniform light intensities. The fluorescent tubes operated at 20 kHz to avoid any interference with the flicker response of the compound eye and the entire setup was covered with black cardboard.^67^ A conventional electrical gear motor (MFA/Como Drills 919D, Conrad, Hirschau, Germany) rotated the stripe drum at various angular velocities. The tested thrips could freely walk in a 30 mm diameter plastic container, covered with a glass lid. The container wall was only ∼5 mm to lower the probability that the animals walked on the container walls. Prior to each experiment, the containers were thoroughly washed with dish soap and cleaned with an anti-static microfiber cloth to minimize electrical forces acting on the walking animal.^68^ For each experiment, we placed the container with one animal in the middle of the arena and immediately started data recording with a video camera (NEC HI-DcamII, Tokyo, Japan) at 60 frames per second, 4 ms image exposure time and 800 pixels x 800 pixels image resolution using video capturing software (Pixoft v3.0, Pixoft Diagnostic Imaging, Birmingham, UK). Total recording time for each run was 68 s.

A direct current motor controls the speed of the rotating arena. A black marker inside the arena was tracked by video at 120 frames per seconds and pattern speed calculated from the number of video frames needed to complete ten 360°-rotations of the visual pattern. The circumference of the circular arena is 0.597 m.

#### Calculation of walking path and spatial corrections

The thrips body was automatically tracked using DLTdv8^69^ and analyzed using a self-written code in MATLAB. Despite the comparatively large diameter of the arena, spatial wavelength of the visual pattern depends on the animal’s position within the 30 mm diameter walking area. Minimum and maximum distance between the animal and the visual pattern was 80 mm and 110 mm, respectively. Thus, the tested wavelengths of 15°, 30°, 45°, 60° and 90° seen from the arena center appear slightly larger at 80 mm (17.8°, 35.3°, 52.4°, 69.9° and 99.8°, respectively) and smaller at 110 mm distance (13.0°, 26.1°, 39.4°, 53.0° and 81.6°, respectively). The rotational velocity is egocentric and calculated from the thrips’ viewpoint at the center of the arena (Figure S2). To consider any difference in mean body position between the different stimulus conditions, we thus determined mean distance of the animal from the arena center in each video frame. We excluded walking trajectories from the statistical analysis (1) in which the thrips exactly followed the circumference of the round container for more than 3 s – the latter indicates that the animals run along the container walls rather than responding to the visual stimulus; (2) if total walk time was less than 10 s. All running traces are, however, shown in Figure 2, even if they were excluded in the analysis. Circular walking trajectories counter to pattern motion was scored as a negative response value. The time traces of the animals’ y-position were broken into pieces defined by two consecutive positive inflection points (Figure 2A iii). The inflection points determine time Δ*t* for one full 360° body rotation during continuous circling. The optomotor response *R*_thrips_ of a thrips was thus calculated as:

Equation 5 $$R_{thrips}=\frac{360{}^{\circ}}{\Delta t}$$

and normalized to maximum *R*_thrips_ measured in each experiment.

### Quantification and statistical analysis

We used self-developed software code written in Matlab® 2018b (The Mathworks) and ImageJ (National Institutes of Health, USA) to process video images, to calculate optical properties and to analyze walking trajectories. The number of tested animals and runs are shown in the figure legends. Throughout the manuscript all data are shown as means ± standard deviation. For graphical representations we used Matlab, Microsoft Powerpoint and CorelDRAW X6 software.
