# Supplementary material for: Miniature brain encodes optomotor response behavior in tiny thrips
Source: iScience. 2026 Feb 10;29(3):114966. doi: 10.1016/j.isci.2026.114966 (PMC12955214; doi:10.1016/j.isci.2026.114966)
Supplement: Document S1. Figures S1–S3 [file mmc1.pdf]

iScience, Volume *29*

Supplemental information

Miniature brain encodes optomotor response behavior in tiny thrips

Tomer Urca and Fritz-Olaf Lehmann

Methods S1: Verification of experimental stimulus condition

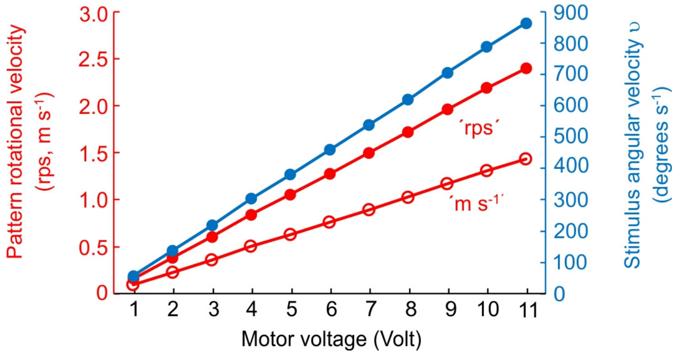

Figure S1. Pattern velocity (red, left scale) was calculated in units of meter per second and rotations per second (rps). Angular velocity of the stimulus (blue, right scale) is shown in unit degrees per second. A direct current motor controls the speed of the rotating arena. A black marker inside the arena was tracked by video at 120 frames per seconds and pattern speed calculated from the number of video frames needed to complete ten 360°-rotations of the visual pattern. The circumference of the circular arena is 0.597 m.

Methods S2: Variance of perceived spatial wavelength and temporal frequency by the animal

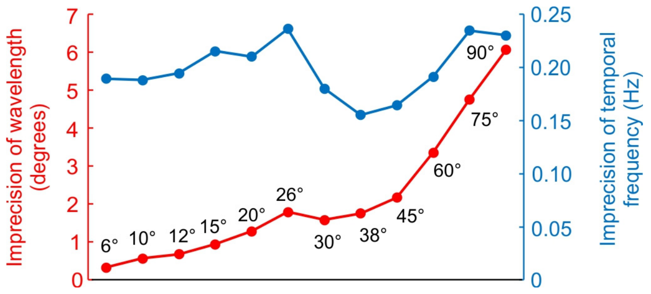

Figure S2. Spatial wavelength and velocity of the rotating pattern are calculated with respect to the center of the arena. As the animals freely walked in a 3 cm diameter Petri dish, their deviations from the arena center leads to deviations from the intended values, in turn causing small changes in the perceived optic flow. Data show standard deviation of the animals' mean position (left scale, wavelength; right scale, temporal frequency of the stimulus with 3.5 Hz) depending on stimulus wavelength λ = 6° – 90°.

Data S3: Changes in the animal's mean turning velocity in response to changes in pattern velocity and wavelength

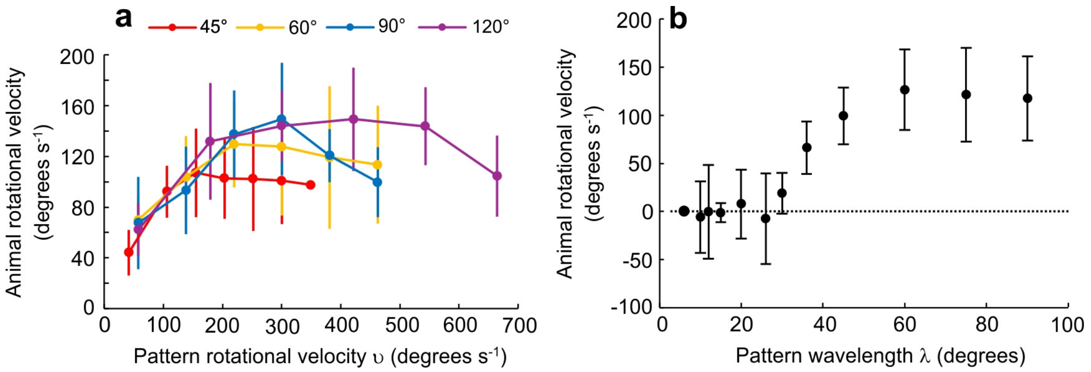

Figure S3. Turning velocity is calculated from the animals' circular paths as explained in the main text. Means ± standard deviation.
